# Conducting Scoping and Systematic Reviews With a Focus on Biocultural Research: The SCRIBE Toolkit

**DOI:** 10.1002/ajhb.70133

**Published:** 2025-09-02

**Authors:** Maria Inês Varela‐Silva, Nathan Rush, Natalie Pearson

**Affiliations:** ^1^ School of Sport, Exercise and Health Sciences Loughborough University Loughborough UK; ^2^ Pilkington Library Loughborough University Loughborough UK

**Keywords:** biocultural methods, Notion, systematic reviews, toolkits, Trello

## Abstract

The SCRIBE (SystematiC Reviews In Biocultural rEsearch) toolkit offers a structured approach for conducting scoping and systematic reviews in biocultural research. It addresses the challenges of synthesizing information, aggregating diverse data, and conducting robust analyses in this field. Biocultural research is vital to anthropology, public health, community health, and policy, as it reveals biological and cultural determinants of health and disparities globally. However, systematic reviews often exclude biocultural factors, leading to biased evidence that overlooks Indigenous, ethnic minority, and small‐scale populations. To fill this gap, the SCRIBE toolkit guides researchers in integrating biocultural frameworks into review methodologies. Developed from a comprehensive literature review and a survey of leading journals (2019–2025), it responds to the underuse of biocultural perspectives in formal reviews. The SCRIBE toolkit is composed of six sequential steps: (i) decide on the type of review, (ii) select a suitable framework (e.g., PICOS, PEO, SPIDER, PCC), (iii) develop the search protocol, (iv) title/abstract screening and full‐text reading, (v) data extraction, risk of bias, and meta‐analysis, (vi) finalize the review. It can be implemented using accessible platforms like Notion and Trello to enhance usability and collaboration or used as a Word file. By mapping biocultural variables into an operational framework and aligning them with established review protocols, the SCRIBE toolkit promotes interdisciplinary, context‐sensitive, and equitable research synthesis. It bridges methodological gaps between anthropology, public health, and evidence‐based practice, supporting the inclusion of marginalized populations and complex cultural contexts. SCRIBE serves as both a practical tool and a call to broaden the epistemological and methodological boundaries of review science within human biology and related fields.

## 1 Background on Biocultural Research

Biocultural research bridges seemingly distinct subfields and employs a wide range of quantitative and qualitative methods. Building on Baker's ecological framework (Baker 1962) subsequent biocultural research has increasingly emphasized the integration of biological, cultural, and environmental factors.

The term “biocultural” was introduced by Hanna and Baker (1979) in their paper, *Biocultural Correlates of Blood Pressure of Samoan Migrants in Hawaii*. Here, the biocultural variable was “place of residence” (rural versus urban) showing an early attempt to link biological health outcomes to cultural and environmental contexts. The field expanded further in the late 1990s with the introduction of political and economic factors (Goodman and Leatherman 1998) that emphasized the role of structural inequities in shaping health outcomes. This marked a shift toward a more critical biocultural framework that integrated insights from political, economic and medical anthropology. Worthman and Kohrt (2005) analyzed how interactions between biological development, cultural practices, and structural conditions shaped health outcomes, emphasizing that enduring health challenges (e.g., chronic disease, mental illness, and infectious disease resurgence) stem from disrupted biocultural dynamics across the life course and require integrated, context‐sensitive interventions. Dufour (2006) traced the historical trajectory of biocultural research, identifying key milestones in the field and addressing several methodological challenges. One key issue was the definition of variables such as socioeconomic status, poverty, and urbanization, which are complex and context‐dependent, requiring careful operationalization. Measuring cultural and social factors posed another difficulty, as biological variables are relatively straightforward to quantify, but cultural factors need ethnographic validation to ensure meaningful measurement. Wiley and Cullin (2016) conducted a bibliometric review of articles published in the American Anthropological Association (AAA) journals and in the American Journal of Human Biology (AJHB), covering the period of 2000–2014. They found that the term “biocultural” was, by then, primarily used to describe the social environment effects on human biology (75% in AAA, 95% in AJHB), with little change in usage over time. The term appeared frequently in health‐related studies but was typically used for framing research rather than for methodological applications and subsequent analysis of the biocultural impacts on the outcome variables. The term was evenly split between biological and cultural anthropology with limited integration between the two. Key recommendations from this bibliometric review included fostering cross‐subfield collaboration through interdisciplinary studies; publishers encouraging researchers to guest edit special journal issues; expanding theoretical approaches by integrating political economy and evolutionary perspectives; engaging with broader interdisciplinary fields such as social epidemiology and public health; and increasing research on bidirectional relationships between biology and culture.

More recent research has, indeed, consistently built upon these initial studies and followed the suggestions of Wiley and Cullin (2016). Two recent special issues of the *American Journal of Human Biology—*“*Continuity and Change in Biocultural Anthropology*” (Hoke and Schell 2020) and “*Biosocial Perspectives on Life in Urban Environments* (Chaney et al. 2025; Schell 2025; Wiley et al. 2025) bring together research showing how the biocultural approach continues to grow and broadened. The special issue of *Continuity and Change in Biocultural Anthropology*” is introduced by Leonard (2020) who highlights how the papers in this issue move beyond early theoretical models to demonstrating practical, mixed‐methods applications of complex biocultural frameworks. He draws attention to the integration of evolutionary and political‐economic perspectives, hypothesis testing in diverse settings, and investigations into the biological impacts of economic and ecological change. The opening article by Hoke and Schell (2020) builds on this by focusing on what biocultural anthropology does, rather than how it is defined. They describe the field's flexibility in integrating evolutionary, ecological, and political‐economic approaches and emphasize its growing engagement with structural violence, inequality, and lived experience. Leatherman and Goodman (2020) revisited and expanded on their original synthesis (Goodman and Leatherman 1998) noting how new developments such as epigenetics, microbiome research, and aspects connected with the Developmental Origins of Health and Disease (DOHaD) support the view that human biology is shaped by social inequalities and local environments. They call for renewed attention to political‐economic structures and advocate for a more critical, integrated, and contextually grounded biocultural anthropology. Wiley (2020) synthesizes insights from the nine papers included in this special issue, pointing to the field's strengths in interdisciplinary collaboration and theoretical adaptability but also calling for greater inclusion of intersectional analysis, qualitative methods, and cultural theory. In this special issue, Brewis and Wutich (2020) propose stigma as a key construct that bridges evolutionary and political‐economic frameworks to explain health disparities. Hicks (2020) uses biocultural models to examine maternal weight and gestational weight gain, showing how social inequalities shape health outcomes. Howells et al. (2020) focus on maternal stress after natural disasters, highlighting the mediating role of social support and economic status in biocultural stress responses. Quinn and Childs (2020) explore how economic development, geography, and environment interact to influence infant growth in high‐altitude Nepal, and Lynn et al. (2020) examined Samoan tattooing as a possible biocultural adaptation linked to immune function, showing how cultural practices may shape biological outcomes.

Other contributions focus on theoretical and applied innovations. Dressler (2020) proposes a three‐dimensional model of culture as an evolutionary niche, aiming to operationalize cultural constructs for biocultural research. Boyette et al. (2020) investigated the impact of fatherhood and egalitarian social structures on child nutrition in Congolese communities, highlighting how caregiving norms shape biological outcomes. Hoke (2020) linked subsistence food production to infant growth in highland Peru, and how economic strategies affect child health. DuBois et al. (2021) extended biocultural research to transgender and gender‐diverse populations, advocating for minimally invasive biomarker use to connect social experiences such as stigma and access to care with physiological and mental health outcomes.

The most recent special issue *Biosocial Perspectives on Life in Urban Environments* focuses on urban settings—one of the earliest focuses of biocultural research—and how these environments shape health and development. Schell (2025) introduced the issue by explaining how urban environments, impacted by political and economic systems, exacerbate new exposures (e.g., pollution, stress, poor diets, and less physical activity) that affect human biology throughout the life course, on a global scale. The editorial framed urbanization not just as a population trend but as a deeply embodied and unequal stressor, with real health consequences. Dervarics et al. (2025) showed how patterns of historic racial segregation in Phoenix, Arizona are still currently linked to poorer health outcomes in affected neighborhoods, highlighting how historical inequalities leave lasting marks on health. Wiley et al. (2025) found that adolescent mothers in Brazil, exposed to violence during pregnancy, showed disrupted cortisol rhythms, in both mothers and infants, revealing intergenerational effects of social stress. Manus et al. (2025) assessed infants in Chicago and found that regular interaction with non‐parental caregivers (alloparents) was linked to greater microbial diversity in skin and stool samples, suggesting that caregiving actions (such as shared feeding or sleeping) impact early microbiome development and are likely to be associated with immune health. Dorsey (2025)'s research on infectious disease in peri‐urban Lima provided evidence that rapid, unequal urban growth, which is associated with poor infrastructures, increases vulnerability to disease. Finally, Chaney et al. (2025) compared contaminant exposures in Argentina and the U.S., finding that the impacts of different chemicals produce similar biological effects on metabolism and cardiovascular risk, suggesting that despite regional differences, human biology responds in consistent ways to environmental toxins.

In summary, biocultural research is complex, multidimensional, and continually evolving. It relies on interdisciplinary collaboration, diverse methods, and considers cultural, political, and environmental contexts to understand human biology and health. These same qualities make scoping and systematic reviews especially challenging, as variations in theories, definitions, and approaches make it difficult to compare and combine findings across studies.

## 2 Background on Scoping and Systematic Reviews

Scoping and systematic reviews are suited to distinct research needs and differ in aims, methods, and outcomes. Scoping reviews explore broad topics, map existing literature, and identify gaps in knowledge without assessing the quality of included studies (Tricco et al. 2016; Tulandi and Suarthana 2021). They are useful when the literature is complex or under‐explored, offering a descriptive overview rather than a detailed and in‐depth synthesis (Munn et al. 2018; Peters et al. 2015). Systematic reviews focus on answering specific research questions by identifying and synthesizing high‐quality evidence using rigorous methods, including quality appraisal. When appropriate, meta‐analyses are used to combine results from multiple independent studies on a topic, identify overall trends, and draw more robust conclusions. Meta‐analyses increase statistical power and can help resolve inconsistencies among individual studies (Peters 2016; Peters et al. 2015; Sucharew 2019). Systematic reviews were first applied in medical sciences in the 1970s (Mallett et al. 2012) and have grown exponentially since the 1980s (Chalmers and Fox 2016) with a more than 20‐fold increase in indexed journal publications over the last 20 years (Hoffmann et al. 2021). For four decades, public health policies worldwide have relied on systematic reviews, which are considered the gold standard for evidence‐based decision‐making in clinical settings and are increasingly used in public health (Chalmers and Fox 2016; Sackett et al. 1996). However, to enhance their impact, systematic reviews should integrate diverse methodologies, including clinical, experimental, mixed‐methods, qualitative, and ethnographic research, while also incorporating top‐down, transversal, and bottom‐up perspectives.

## 3 Methodological Rationale for Developing the SCRIBE Toolkit

We hypothesized that there is relatively limited inclusion of biocultural factors in public health scoping and systematic reviews and that this leads to biases, as often public health reviews exclude indigenous, ethnic minority, and small‐scale populations central to biocultural research. Furthermore, we hypothesized that within the main Human Biology/Biocultural journals, scoping and systematic reviews are not the preferred research output. To test these assumptions, we assessed eight relevant journals. We defined the timeframe between 2019 and 2025, accounting for the increase in reviews published during the Covid pandemic. Our main three journals of interest were: The American Journal of Human Biology (AJHB), the Annals of Human Biology (AHB) and the American Journal of Physical/Biological Anthropology (AJBA). Then, we assessed five other journals identified as being similar to the AJHB according to the Scimago Journal Rankings (https://www.scimagojr.com/) that, however, tended to include a great deal more epidemiology and public health research. This identification selected: Plos One, Proceedings of the National Academy of Sciences (PNAS), BMC Public Health, Medical Anthropology Quarterly, Anthropologischer Anzeiger, Medical Anthropology: Cross‐Cultural Studies in Health and Illness, Ecology and Society, Journal of Ethnobiology, and Journal of Ethnobiology and Ethnomedicine.

The Scopus database was then used to identify: (i) the total number of outputs in each journal, within the set timeframe; (ii) how many of these were systematic or scoping reviews; (iii) how many were other types of reviews (i.e., umbrella, mapping reviews) and (iv) how many, explicitly, used the term “biocultural”. The findings are shown in Table 1.

**TABLE 1:** Overview of review types and use of the term “Biocultural” in selected journals (based on the Scopus database).

Journal	Total number of articles (2019–2025)	Scoping/Systematic reviews	% Scoping//Systematic reviews	Term “Biocultural” mentioned in scoping or systematic Reviews	Other types of reviews
American Journal of Human Biology (AJHB)	899	9 (2 Scoping +7 Systematic)	1.00%	0	2 Critical, 1 Multidisciplinary
Annals of Human Biology (AHB)	401	6 (Systematic)	1.50%	0	1 Non‐systematic
American Journal of Biological Anthropology (AJPA/AJBA)	1059	1 (Systematic)	0.09%	0	1 Dataset Review
PLOS ONE	101 440	1285 (Systematic)	1.27%	0	6 Rapid Scoping, 5 Rapid Systematic, 12 Overview, 1 Critical, 4 Rapid, 6 Realist, 14 Umbrella
Proceedings of the National Academy of Sciences (PNAS)	24 742	1 (Systematic)	0.004%	0	0
BMC Public Health	15 159	207 (40 Scoping +167 Systematic)	1.37%	0	3 Umbrella, 1 Rapid Systematic, 1 Rapid Realist, 1 Lexical, 1 Interpretive Synthesis
Medical Anthropology Quarterly	197	0	0%	0	0
Anthropologischer Anzeiger	222	1 (Systematic)	0.45%	1	0
Medical Anthropology: Cross Cultural Studies	385	0	0%	0	0
Ecology and Society	889	0	0%	0	0
Journal of Ethnobiology	195	0	0%	0	0
Journal of Ethnobiology and Ethnomedicine	469	4 (Systematic)	0.85%	2	0

PLOS ONE had the highest absolute number of systematic reviews, publishing a total of 1285 between 2019 and 2025. This is consistent with its broad publishing scope and high article volume, which accommodates a larger number of review‐type publications. In contrast, when considering the proportion of scoping/systematic reviews relative to total output, the Annals of Human Biology had the highest percentage at 1.5%, followed by BMC Public Health at 1.37%. Despite the presence of scoping and systematic reviews across several journals, the use of the term “Biocultural” in such reviews was extremely rare, appearing only three times in total. Specifically, there was one mention in Anthropologischer Anzeiger and two in the Journal of Ethnobiology and Ethnomedicine, revealing the limited integration of biocultural frameworks within formal review literature during this period. The Medical Anthropology Quarterly, Medical Anthropology: Cross Cultural Studies, Ecology and Society, and the Journal of Ethnobiology did not publish any scoping or systematic reviews. Finally, both PLOS ONE and BMC Public Health published other types of reviews that use broader methodological approaches in evidence synthesis. The American Journal of Human Biology published critical reviews and a multidisciplinary review, the Annals of Human Biology included a non‐systematic review, and the American Journal of Biological Anthropology published a dataset review.

Given the minimal number of explicit references on “biocultural” perspectives in existing scoping and systematic reviews, there is a clear gap in the synthesis of research. Biocultural frameworks are central to understanding the complex interactions between biology, culture, and environment. Therefore, their integration into evidence synthesis is important for developing a more comprehensive and context‐sensitive understanding of health, human behavior, and social processes. Expanding the use of biocultural‐based scoping and systematic reviews would not only address this gap but also further support the development of interdisciplinary research to better inform practice and policy.

## 4 The Role of Digital Systems and Tools in Managing Scoping and Systematic Reviews

Conducting scoping or systematic reviews is inherently complex, requiring researchers to navigate a vast array of tools, guidelines, and quality control frameworks to ensure rigor and reproducibility (e.g., Harris et al. 2014; Methley et al. 2014; Page et al. 2021). Traditional methods relying solely on Excel or Word files can quickly become overwhelming, as these lack the necessary structure for efficiently tracking study selection, data extraction, and critical appraisal across multiple reviewers and bibliographic databases. To address these challenges, the SCRIBE toolkit is here implemented through Notion and Trello, two widely used digital platforms for organization and project management that, despite their versatility that saves a great deal of time and effort, are still very underused (Castro‐Arquinigo et al. 2023; Kaur 2018; Rysavy and Michalak 2020). Table 2 shows the main characteristics of both platforms.

**TABLE 2:** Main features of Notion and Trello.

Feature	Notion	Trello
Structure and core functionality	A highly flexible all‐in‐one workspace that combines notetaking, databases, wikis, and task management. Supports interconnected pages, databases, and kanban boards.	Primarily a kanban‐based task management tool, focusing on boards, lists, and cards for workflow visualization. Ideal for simple, linear task tracking and agile methodologies.
Customization and flexibility	Highly customizable with support for databases, embedded content, and various display formats (tables, lists, calendars, galleries). Can function as a knowledge base or task tracker.	More streamlined, with limited customization beyond boards and cards. Supports labels, checklists, and automation but lacks deep database functionality.
Collaboration and teamwork	Best for teams needing document collaboration alongside task management. Allows task assignment, comments, and centralized knowledge management.	Best for teams needing simple task‐tracking with a clear progress visualization. Excellent in kanban‐style workflows (e.g., “To Do,” “In Progress,” “Done”) but lacks deeper document management features.
Learning curve and usability	Steeper learning curve due to vast customization options and database functionality. Best for users who need an all‐in‐one tool and are willing to invest time in setup.	Intuitive and easy to use from the start. Best for teams needing a straightforward, visual task management system with minimal setup.

## 5 How to Use the SCRIBE Toolkit

The SCRIBE toolkit is composed of six steps. Each step contains tasks in a sequential but inter‐related order. Each task contains a to‐do list where actions can be ticked off once they are completed. The SCRIBE toolkit is available in multiple formats: a Notion page, a Trello board, and a Word file template (Supporting Information 1), all accessible via https://doi.org/10.17028/rd.lboro.29364935.

An associated workbook (Supporting Information 2) is also provided to help authors organize their ideas and to develop inclusion/exclusion criteria and search strategies. The implementation of SCRIBE is straightforward: (i) choose which format to use (i.e., the Notion page, the Trello board or the Word template); (ii) download the SCRIBE; and (iii) follow the template sequentially. Authors are strongly advised not to skip steps, tasks, or to‐dos.

## 6 Conclusion

The SCRIBE toolkit helps researchers include biocultural perspectives in scoping and systematic reviews, where they are often missing. It provides a clear, step‐by‐step process that makes complex reviews easier to manage and more inclusive. By using platforms like Notion and Trello, the SCRIBE toolkit is easy to access and use. Overall, it supports high quality and more balanced research that better systematizes research focused on human variation across diverse settings, worldwide.

## Author Contributions

**Maria Inês Varela‐Silva:** conceptualization (lead), Investigation, Formal Analysis, Methodology, Project Administration, Visualization and writing (Original draft preparation). **Nathan Rush:** data curation, formal analysis, investigation, writing (Review and editing). **Natalie Pearson:** investigation, Formal Analysis, Methodology, Validation, Writing (Review and Editing).

## Ethics Statement

The authors have nothing to report.

## Conflicts of Interest

The authors declare no conflicts of interest.

## Supporting information


**Data S1:** Supporting Information.

**Data S2:** Supporting Information.

## Data Availability

The data that support the findings of this study are openly available in Loughborough University Research Repository at https://repository.lboro.ac.uk/articles/online_resource/The_SCRIBE_toolkit/29364935.
