# Supplementary material for: Conducting Scoping and Systematic Reviews With a Focus on Biocultural Research: The SCRIBE Toolkit
Source: Am J Hum Biol. 2025 Sep 2;37(9):e70133. doi: 10.1002/ajhb.70133 (PMC12402747; doi:10.1002/ajhb.70133)
Supplement: Supplementary file 1 — Data S1: Supporting Information. [file AJHB-37-e70133-s001.dotx]

**
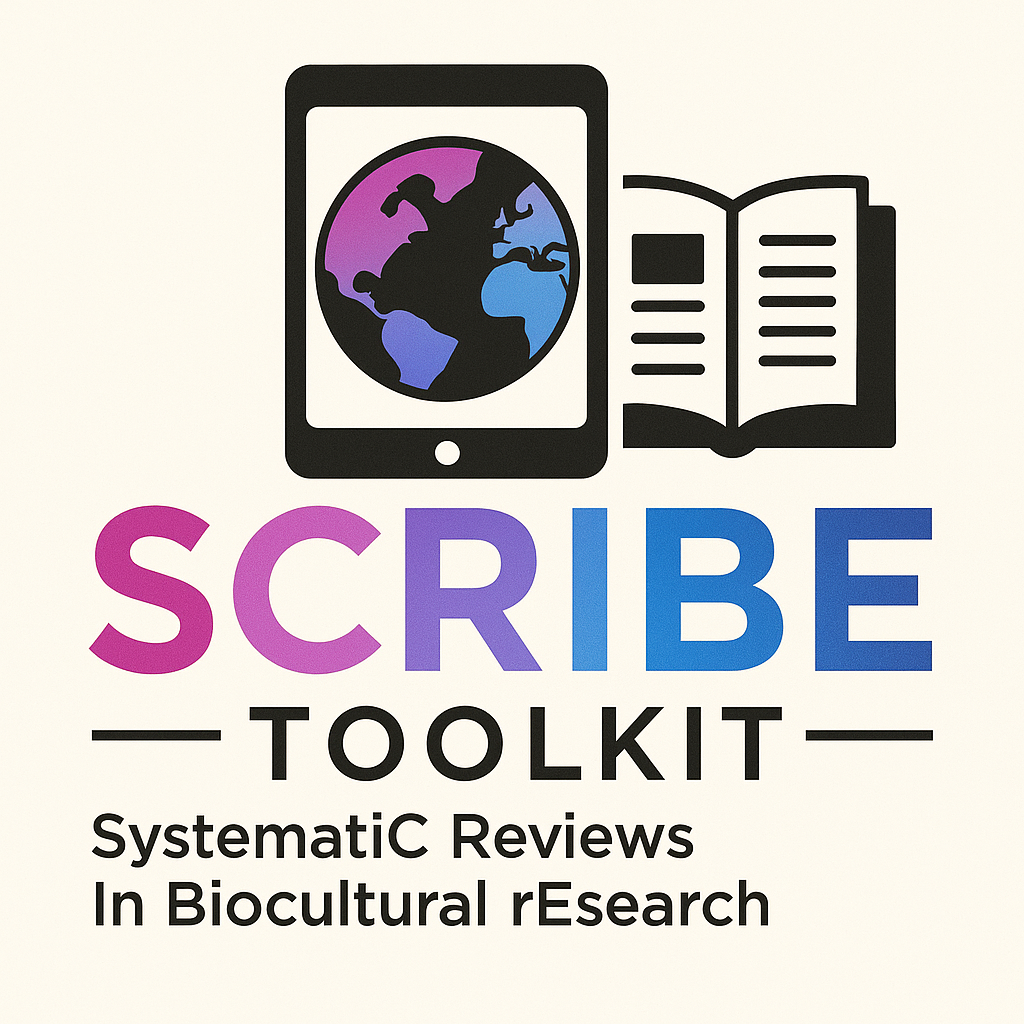
**

**How to cite this toolkit:** Varela-Silva, M.I., Rush, E. and Pearson, A.L. (2025). Conducting scoping and systematic reviews with a focus on biocultural research: The SCRIBE toolkit. *American Journal of Human Biology*. doi ADD DOI

**SCRIBE WORKBOOK**

**A. Develop your research question**

*Use this space to jot down ideas and explore different options that will help shape a well-developed and thoroughly considered research question.*

**B. Research question refined**

*Use this space to write the final version of your question.*

1. **Based on the research question from point B, identify and extract all relevant keywords. List each keyword here and provide a clear definition supported by an appropriate citation.**

KW1.

KW2.

KW3.

KW4.

KW5.

KW6.

1. **Identify synonyms for each keyword, verify them using** [**PubMed**](https://pubmed.ncbi.nlm.nih.gov/)**, and indicate which are recognized** [**MeSH**](https://www.ncbi.nlm.nih.gov/mesh/) **(Medical Subject Headings) terms.**

|  | **Keywords** | **Synonyms** | **Mesh term (Y/N)*** |
| --- | --- | --- | --- |
| **KW1** |  |  |  |
|  |  |  |  |
|  |  |  |  |
|  |  |  |  |
|  |  |  |  |
|  |  |  |  |
|  |  |  |  |
| **KW2** |  |  |  |
|  |  |  |  |
|  |  |  |  |
|  |  |  |  |
|  |  |  |  |
|  |  |  |  |
|  |  |  |  |
|  |  |  |  |
| **KW3** |  |  |  |
|  |  |  |  |
|  |  |  |  |
|  |  |  |  |
|  |  |  |  |
|  |  |  |  |
|  |  |  |  |
|  |  |  |  |
| **KW4** |  |  |  |
|  |  |  |  |
|  |  |  |  |
|  |  |  |  |
|  |  |  |  |
|  |  |  |  |
|  |  |  |  |
| **KW5** |  |  |  |
|  |  |  |  |
|  |  |  |  |
|  |  |  |  |
|  |  |  |  |
|  |  |  |  |
|  |  |  |  |
| **KW6** |  |  |  |
|  |  |  |  |
|  |  |  |  |
|  |  |  |  |
|  |  |  |  |
|  |  |  |  |
|  |  |  |  |

***** Any keyword or synonym that is not a MeSH term should be classified as a 'text word'

1. **Organize keywords, MeSH terms and text words according to the framework that better suits your needs (e.g. PICOS; PEO/PECO; SPiDER, PCC)**

|  | **P** | **I** | **C** | **O** | **S** |
| --- | --- | --- | --- | --- | --- |
| **Keywords** |  |  |  |  |  |
| **MeSH Terms** |  |  |  |  |  |
| **Text Words** |  |  |  |  |  |

P (Patient, problem or population); I (Intervention, prognostic factor, exposure); C (comparative intervention, comparison); O (Outcome); S (Studies, types of studies).

|  | **P** | **E** | **O** | **C**  **(optional)** |
| --- | --- | --- | --- | --- |
| **Keywords** |  |  |  |  |
| **MeSH Terms** |  |  |  |  |
| **Text words** |  |  |  |  |

P (Patient, population); E (exposure); O (Outcome); C comparison)

|  | **S** | **P** | **I** | **D** | **E** | **R** |
| --- | --- | --- | --- | --- | --- | --- |
| **Keywords** |  |  |  |  |  |  |
| **MeSH Terms** |  |  |  |  |  |  |
| **Text Words** |  |  |  |  |  |  |

SPIDER: (Sample, Phenomenon of Interest, Design, Evaluation, Research type)

|  | **P** | **C** | **C** |
| --- | --- | --- | --- |
| **Keywords** |  |  |  |
| **MeSH Terms** |  |  |  |
| **Text Words** |  |  |  |

PCC: Population, Concept, Context

1. **Define inclusion/exclusion criteria based on the chosen framework**

|  | **Inclusion** | **Exclusion** |
| --- | --- | --- |
| **PICOS** |  |  |
| P (population, patient) |  |  |
| I (Intervention, factor) |  |  |
| C (Comparison) |  |  |
| O (Outcome) |  |  |
| S (Types of Studies) |  |  |
| **PEO/PECO** |  |  |
| P (population, patient) |  |  |
| E (exposure) |  |  |
| C (Comparison) |  |  |
| O (Outcome) |  |  |
| **SPIDER** |  |  |
| S (Sample) |  |  |
| Pi (Phenomenon of interest) |  |  |
| D (Design) |  |  |
| E (Evaluation) |  |  |
| R (Research Type) |  |  |
| **PCC** |  |  |
| P (Population) |  |  |
| C (Concept) |  |  |
| C (Context) |  |  |

1. **Data extraction tables templates**

These tables can be easily structured in Excel, Word, or directly within several systematic review software platforms. Add a row for each study, and include additional columns as needed to extract other relevant variables.

**Table 1. Study characteristics and ethics information**

| **Study ID / Authors / Year / Country** | **Study Design** | **Sample Size** | **Setting** | **Intervention / Comparator Details** | **Funding Source** | **Ethical Approval / Ethics Committee / Informed Consent** |
| --- | --- | --- | --- | --- | --- | --- |
|  |  |  |  |  |  |  |

**Study ID, Authors, Year, Country:** Basic identifying information for each study.

**Study Design:** Type of study (e.g., randomized controlled trial, cohort study).

**Sample Size:** Total number of participants.

**Setting:** Description of study setting (e.g., community, urban/rural, hospital).

**Intervention/Comparator Details:** Brief descriptions of the intervention and comparator.

**Funding Source:** Information on funding or conflicts of interest.

**Ethical Approval, Ethics Committee, Informed Consent:** Details on ethics processes.

**Table 2. Sample characteristics and biocultural variables**

| **Study ID** | **Age (Mean ± SD)** | **Sex/Gender Distribution** | **Ethnicity/Race** | **Socioeconomic Status** | **Education Level** | **Add more variables as needed** |
| --- | --- | --- | --- | --- | --- | --- |
|  |  |  |  |  |  |  |

**Study ID:** Identifier for the study.

**Age (Mean ± SD):** Mean age and standard deviation, or age range if available.

**Sex/Gender Distribution:** Percentage or count by sex/gender.

**Biocultural characteristics relevant to the study:** Ethnicity/Race, Socioeconomic Status, Education Level. These are examples only. Authors should define their biocultural variables of interest in the methods’ section and adapt this table accordingly.

**Table 3. Outcome Analysis with Biocultural Context, Meta-Analysis Data, and Quality/Risk of Bias Summary**

| **Study ID** | **Outcome Measured** | **Outcome Type**  **(Continuous / Dichotomous)** | **Intervention Group Outcome**  **(Mean ± SD or Events/N)** | **Comparator Group Outcome (Mean ± SD or Events/N)** | **Effect Size (CI)** | **p-value** | **Heterogeneity (I²)** | **Biocultural Impact Summary** | **Quality Control / Risk of Bias** |
| --- | --- | --- | --- | --- | --- | --- | --- | --- | --- |
|  |  |  |  |  |  |  |  |  |  |

**Outcome Measured, Outcome Type:** Description of the primary outcome, indicating whether it is continuous or dichotomous.

**Intervention/Comparator Group Outcome:** Report outcome data for intervention and control groups (when applicable).

**Effect Size (CI), p-value:** Effect size with confidence intervals and statistical significance (when applicable).

**Heterogeneity (I²):** Heterogeneity measure if data is suitable for meta-analysis.

**Biocultural Impact Summary:** Summary of how biocultural factors influenced the outcome.

**Quality Control/Risk of Bias:** Summary of studies’s quality control and risk of bias assessment, providing an overall result. Different quality-control/RoB tools have their own assessment process, and this column should reflect the summary. Details of the entire Quality Control/Risk of Bias process should be shown in the supplemental materials of your review.

**Table 4. Suggestions for Quality Control/ROB tools**

| Tool Name | Study Type | Format | Key Criteria/Features | Reference |
| --- | --- | --- | --- | --- |
| Cochrane Risk of Bias Tool 2 (RoB 2) | RCTs | Domain-based | Assesses bias in 5 domains including randomization, deviations from interventions, and outcome measurement; outputs Low/Some concerns/High risk. | [Sterne et al., 2019 (BMJ)](https://doi.org/10.1136/bmj.l4898) |
| Jadad Scale | RCTs | Scoring Scale | 3-item scale (randomization, blinding, withdrawals); total score out of 5. | [Jadad et al., 1996](https://pubmed.ncbi.nlm.nih.gov/8721797/) |
| CASP RCT Checklist | RCTs | Checklist (narrative) | 11 question-based items assessing validity, results, and applicability; no scoring. | [CASP UK (2018)](https://casp-uk.net/casp-tools-checklists/) |
| Newcastle–Ottawa Scale (NOS) | Observational (Cohort, Case-Control) | Scoring System | Star-based system assessing selection, comparability, and outcome/exposure; max 9 stars. | [Wells et al., 2011](https://www.ohri.ca/programs/clinical_epidemiology/oxford.asp) |
| ROBINS-I | Non-randomized studies of interventions | Domain-based | Assesses 7 bias domains using signaling questions; judgment ranges from Low to Critical risk. | [Sterne et al., 2016](https://pubmed.ncbi.nlm.nih.gov/27733354/) |
| MINORS | Non-randomized studies | Scoring Checklist | 12 items scored 0-2; covers methodology and reporting quality; max 24 for comparative studies. | [Slim et al., 2003](https://pubmed.ncbi.nlm.nih.gov/12956787/) |
| Kmet Standard Quality Assessment Criteria | Quantitative and Qualitative | Scoring Checklists | Separate 14- and 10-item checklists; scores as percentage; adaptable across study types. | [Kmet, Lee, & Cook, 2004 (AHFMR)](https://era.library.ualberta.ca/items/48b9b989-c221-4df6-9e35-af782082280e) |
