# Supplementary material for: Conducting Scoping and Systematic Reviews With a Focus on Biocultural Research: The SCRIBE Toolkit
Source: Am J Hum Biol. 2025 Sep 2;37(9):e70133. doi: 10.1002/ajhb.70133 (PMC12402747; doi:10.1002/ajhb.70133)
Supplement: Supplementary file 2 — Data S2: Supporting Information. [file AJHB-37-e70133-s002.dotx]

## Step 1: Decide on the Type of Review

| ✔ | **Tasks** |
| --- | --- |
| ☐ | **1.1 Develop and refine your research question.** Clearly articulate your research question to define the scope and focus of your investigation. Justify its significance by situating it within the existing literature, referencing key studies to identify current knowledge gaps (use sections A and B of the workbook). |
| ☐ | **1.2 Decide on whether to do a scoping or systematic review**. Read the following articles and decide which is the most suitable type of review for your research question.   - Grant & Booth (2009): [LINK](https://onlinelibrary.wiley.com/doi/10.1111/j.1471-1842.2009.00848.x) - Peters (2020) : [LINK](https://journals.lww.com/jbisrir/fulltext/2020/10000/updated_methodological_guidance_for_the_conduct_of.4.aspx) - Higgins *et al.* (2022) : [LINK](https://training.cochrane.org/handbook) |
| ☐ | **1.3 Write rationale for the type of review.** Once you have decided whether to conduct a Scoping Review (ScR) or a Systematic Review (SR), write a paragraph justifying your choice, clearly explaining the rationale and ensuring that all sources are appropriately cited. |
| ☐ | **1.4 Communicate with co-authors.**   - Confirm and clearly define roles and responsibilities with all co-authors. - Select a target journal and review its authors’ guidelines thoroughly. - Ensure all co-authors have reviewed and provided feedback on each component of Step 1. |

## Step 2: Select the Appropriate Framework

| ✔ | **Tasks** |
| --- | --- |
| ☐ | **2.1 Select a suitable framework (PICOS, PEO/PECO, SPIDER, PCC).**   - *PICOS* (Population/Patient/Problem, Intervention/Exposure, Comparison, Outcome, and Study design) is a framework traditionally used to structure research and review questions focused on interventions (Methley *et al.*, 2014). Although primarily applied to intervention studies, PICOS can be adapted for observational research. In such cases, the 'I' may refer to an exposure or factor of interest rather than a purposeful intervention, and the 'C' may represent a natural comparison group (e.g., exposed vs. unexposed), rather than a predefined comparator. However, alternative frameworks, such as PEO, PECO or SPIDER, may be more suitable for observational or qualitative studies. - *PEO/PECO* (Population, Exposure, Outcome, and optional Comparator) is well-suited for epidemiological and qualitative research, particularly in cases where exposures - rather than interventions - are the primary focus (Saaiq and Ashraf, 2017). - *SPIDER* (Sample, Phenomenon of Interest, Design, Evaluation, Research type) was developed for qualitative and mixed-methods reviews. It removes the need for intervention or comparison components by emphasizing the phenomenon of interest, study design, and type of research (Methley *et al.*, 2014). - *PCC* (Population, Concept, Context) was developed by the The Joanna Briggs Institute (2015) for use in scoping reviews. It is designed to support broad, exploratory research questions without requiring an intervention component. |
| ☐ | **2.2 Plan your search: keywords, synonyms, MeSH/text words, criteria.**   - Identify and extract all relevant keywords from your refined research question. List each keyword and provide a clear definition supported by appropriate citations (Use section C of the workbook). - For each keyword, identify relevant synonyms and determine which are MeSH terms and which are text words (Use section D of the workbook). - Organize keywords, MeSH terms and text words according to the framework that better suits your needs (Use section E of the workbook). - Define inclusion/exclusion criteria based on the chosen framework (Use section F of the workbook). |
| ☐ | **2.3 Communicate with co-authors.**   - Ensure all co-authors have reviewed and provided feedback on each component of Step 2 |

## Step 3: The Search Protocol

| ✔ | **Tasks** |
| --- | --- |
| ☐ | **3.1 Build search strategy in PubMed.**   - Create an account or login [to PubMed](https://pubmed.ncbi.nlm.nih.gov/). - Build the Search Strategy on PubMed, combining your Keywords, MeSH terms, and text words by conducting separate searches for each component of the framework of your choice (PICOS, PEO/PECO, SPIDER, and PCC) using the “OR” Boolean operator and the “AND” Bolean operator for the final search.   *Example for PICO.*   1. *Search #1: all keywords, MeSH terms and text words identified in section E of the workbook for P (participants), connected with the “OR” Boolean operator.* 2. *Search #2: all keywords, MeSH terms and text words identified in section E of the workbook for I (Intervention), connected with the “OR” Boolean operator.* 3. *Search #3: all keywords, MeSH terms and text words identified in section E of the workbook for C (comparison), connected with the “OR” Boolean operator.* 4. *Search #4: all keywords, MeSH terms and text words identified in section E of the workbook for O (outcomes), connected with the “OR” Boolean operator.* 5. *Combine the searches using the "AND" Boolean operator (e.g., #1 AND #2 AND #3 AND #4).*  - Run the search. |
| ☐ | **3.2 Run and save searches in other databases.**   - Run similar searches on other bibliographic databases (e.g Scopus, Web of Science, EMBASE, Cochrane). - Save each database search in an appropriate format (e.g., CSV, RIS, XML, or PubMed.txt), as these files will be required for upload and articles’ management for screening and full-text review in Step 4-Task 4.2. - Set up regular alerts for new publications.   Agree on a firm date to stop the searches. |
| ☐ | **3.3 Communicate with co-authors.**   - Ensure all co-authors agree with the search strategy. - Agree on next steps. - Confirm roles and responsibilities. |
| ☐ | **3.4 Review PRISMA guidelines and EQUATOR checklists**. The PRISMA (Preferred Reporting Items for Systematic Reviews and Meta-Analyses) guidelines are a set of evidence-based recommendations for reporting systematic reviews and meta-analyses to ensure clarity, transparency, and completeness. There are several PRISMA extensions tailored for different types of reviews and reporting needs. For example, for scoping reviews the PRISMA-ScR extension should be used.   - Read the [PRISMA 2020](https://www.equator-network.org/reporting-guidelines/prisma/) statement - Get acquainted with the [EQUATOR Network](https://www.equator-network.org/reporting-guidelines/prisma/) (2024). - Select relevant checklists and references. |
| ☐ | **3.5 Submit to PROSPERO.** PROSPERO is an international database for prospectively registering systematic reviews in health and social care. It is maintained by the University of York's Centre for Reviews and Dissemination. The main goal of PROSPERO is to promote transparency, reduce duplication, and minimize bias in systematic review research by making protocols publicly available before the reviews are conducted.   - Register or log in to [PROSPERO](https://www.crd.york.ac.uk/prospero/). - Ensure the review meets PROSPERO eligibility criteria. - Prepare the protocol, detailing objectives, methods, search strategy, and analysis plans following the PROSPERO form. - Confirm with co-authors - Submit PROSPERO application. |
| ☐ | **3.6 Choose and set up reference management software (e.g. RefWorks, EndNote, Mendeley, Zotero)**   - Sign up or log in. - Install the appropriate "cite and write" plug-in for your Word processor. |
| ☐ | **3.7 Review and consolidate all work: draft intro, methods, analysis.**   - Open a Word file and format it according to the target journal's author guidelines. - Draft the introduction section:   - introduce the topic,   - explain its relevance,   - summarise what is already known,   - identify gaps, inconsistencies or controversies,   - explain why the review is needed,   - clarify the potential contribution of the review to the field,   - state the main aim,   - include research questions (or hypothesis if applicable). - Draft the Methods section:   - Describe the steps of this toolkit as a guide for the development of your methods. Take enough time to ensure it is clear, consistent, and well-structured.   - Define how you will analyse the extracted data, based on your research question.   - If meta-analysis is not feasible due to heterogeneity of outcomes, consider other options such as thematic analysis (Braun and Clarke, 2019; Campbell *et al.*, 2021), narrative synthesis (Popay *et al.*, 2006), framework synthesis (Brunton, Oliver and Thomas, 2020) or critical appraisal (Tod, Booth and Smith, 2022). Even if meta-analysis is feasible, these narratives are likely to strengthen the biocultural context and further support meta-analysis results. |
| ☐ | **3.8 Communicate with co-authors.**   - Ensure all co-authors have reviewed and provided feedback on each component of Step 3. |

## Step 4: Title/Abstract Screening and Full-Text Reading

| ✔ | **Tasks** |
| --- | --- |
| ☐ | **4.1 Set up screening platform (e.g. Covidence or Rayyan).** This should be able to deal with screening, organization, and management of studies; application of inclusion and exclusion criteria; collaboration with team members; and tracking of decisions throughout the workflow. Here we suggest the use of either [Covidence](http://www.covidence.org/) (paid software), or [Rayyan](https://www.rayyan.ai/) (free software).   - Create an account or log in to Covidence or Rayyan. - Follow the on-screen instructions to set up your review dashboard. - Complete all required fields and respond to all prompts, including optional actions, where applicable. - Detail your inclusion/exclusion criteria (Use section F of the workbook). |
| ☐ | **4.2 Import search results and remove duplicates.**   - Import the search files gathered in Task 3.2. into the software. - Ensure that the software's 'Check for Duplicates' feature is enabled. |
| ☐ | **4.3 Assign co-author roles.**   - Agree on specific roles for title/abstract screening, full-text reading, data extraction, and risk of bias (RoB) assessment among co-authors. |
| ☐ | **4.4 Title/Abstract screening and PRISMA chart update.**   - Read the title and the abstract of all papers and exclude them if they do not meet the inclusion criteria. Pass them to the “full text” stage if they provide enough information for you to be confident that they meet your inclusion criteria, or if they only provide insufficient information for you to be certain. At this stage you do not need to provide justifications for excluding papers. - Title and abstract screening can be done by single screeners, with a minimum of 20% of the excluded papers checked by an independent co-author (i.e. someone who has not screened the titles and abstracts). - Ensure that the PRISMA flow chart is being automatically updated in the software. |
| ☐ | **4.5 Full-text reading and reason recording.**   - Access all full-text references and download the PDFs. - Import the full texts into both your review software (e.g., Covidence or Rayyan) and your reference management system (e.g., RefWorks, EndNote, Mendeley, Zotero). - Contact your library liaison if you need assistance obtaining full-text PDFs. - Assign two independent reviewers (co-authors) to assess each full text against the inclusion criteria. - Record reasons for exclusion at the full-text screening stage (e.g., ineligible population, unsuitable study design, or lack of relevant outcomes) using your review software. - Update the PRISMA flow chart as needed, although this should be done automatically by the software. |
| ☐ | **4.6 Communicate with co-authors**   - **Ensure all co-authors have reviewed and provided feedback on each component of Step 4.** |

## Step 5: Data Extraction, RoB, and Meta-Analysis

| ✔ | **Tasks** |
| --- | --- |
| ☐ | **5.1 Create or customize data extraction sheets**   - Based on your research question, create data extraction sheets to systematically identify and collect all relevant information from each study. - If the data meet meta-analysis requirements, extract the necessary information at this stage and gather data pertinent to assessing the risk of bias, as well. - Review each paper thoroughly and extract all essential data in a single, organized effort.   Section G of the workbook provides guidance on building data extraction tables in excel or customizing data extraction templates accessible in the software. It also suggests a range of tools for quality control and risk of bias (RoB) assessment. |
| ☐ | **5.2 Draft narratives for tables**   - Draft the narrative for each table. At this stage, it is sufficient to capture the main points of each table, even if the writing is not fully polished. |
| ☐ | **5.3 Conduct meta-analysis (if applicable)**   - If the extracted data allows, conduct a meta-analysis. Choose suitable software, such as RevMan, Comprehensive Meta-Analysis (CMA), STATA, R, Meta XL, or Open Meta (Brown University), and proceed with the analysis. |
| ☐ | 5.4 Communicate with co-authors   - Ensure all co-authors have reviewed and provided feedback on each component of Step 5. |

## Step 6: Finalize the Review

| ✔ | **Tasks** |
| --- | --- |
| ☐ | **6.1 Confirm all sections are drafted and aligned with journal and PRISMA**   - Ensure that the main document includes advanced drafts of all sections. - Each table or figure in the methods and results should have, at this point, an advanced draft of its narrative, including meta-analysis if applicable. - Refer to the PRISMA checklist to confirm that all information is in the correct sections. - Confirm the manuscript is according to the targeted journal’s guidelines for authors. - Produce the final document. - Send to co-authors for reviews and final round of suggestions. |
| ☐ | **6.2 Submit to the selected journal 🚀** |
| ☐ | **6.3 Celebrate 🎉** |

**NOTES**
